# The effect of indirect moxibustion with the treatment of Ma Ren Pill on patients with acute exacerbation of chronic obstructive pulmonary disease with constipation

**DOI:** 10.3389/fmed.2026.1741212

**Published:** 2026-08-18

**Authors:** Yangdi Peng, Weihui Chen, Qin Wang, Zhuyin Zhou, Shumin Guan, Qiongxu Ye, Lebin Lu, Bangman Zhang, Bin Chen

**Affiliations:** Department of Respiratory Medicine, Yongjia County Traditional Chinese Medicine Hospital, Wenzhou, China

**Keywords:** acute exacerbation, chronic obstructive pulmonary disease, constipation, indirect moxibustion, Ma Ren Pill

## Abstract

**Objective:**

This study aims to explore the effects of indirect moxibustion combined with Ma Ren Pill on the time to resolution of constipation and respiratory symptoms, hospitalization duration, lung function, blood gas indicators, inflammatory factors, clinical efficacy, quality of life, and the incidence of adverse reactions in patients with acute exacerbation of chronic obstructive pulmonary disease (AECOPD) accompanied by constipation.

**Methods:**

A total of 102 patients with AECOPD and constipation who received treatment at our hospital between March 2023 and March 2024 were included in this retrospective study. They were divided into two groups based on the treatment modalities recorded in the electronic medical record system: the control group (*n* = 49 treated with Ma Ren Pill) and the observation group (*n* = 53; treated with indirect moxibustion plus Ma Ren Pill). The times to recovery from constipation and respiratory symptoms, pulmonary function parameters (including forced expiratory volume in the first second (FEV_1_), peak expiratory flow rate (PEF), and forced expiratory volume in the first second as a percentage of forced vital capacity (FEV_1_/FVC)), blood gas indices (partial pressure of oxygen [PO$_2$] and arterial partial pressure of carbon dioxide [PCO$_2$]), inflammatory markers (interleukin-8[IL-8] tumor necrosis factor-alpha (TNF-*α*), and (CRP)), clinical efficacy, quality of life (assessed using the Comprehensive Quality of Life Assessment Questionnaire (GQOLI-74)), and the incidence of adverse reactions were evaluated and compared between the two groups.

**Results:**

After treatment, the observation group showed significantly shorter times to resolution of constipation and shorter hospital stays, as well as significantly lower constipation symptom scores, respiratory symptom scores, and PCO₂, TNF-*α*, IL-8, and CRP levels compared with the control group, with statistically significant differences between the groups (*p* < 0.05). Additionally, the observation group had significantly higher PEF, FEV₁, FEV₁/FVC, PO₂, and quality of life scores than the control group (*p* < 0.05). The total clinical effectiveness rate in the observation group was 98.11%, which was significantly higher than that of the control group’s 83.67% (*p* < 0.05). The overall incidence of adverse reactions did not differ significantly between the two groups (7.55% vs. 4.08%, *p* = 0.457).

**Conclusion:**

Indirect moxibustion combined with Ma Ren Pill was associated with a shorter time to resolution of constipation. Furthermore, this regimen was associated with improved lung function, blood gas indices, quality of life, respiratory and constipation symptom severity, and inflammatory responses, while maintaining an acceptable safety profile. However, a causal relationship cannot be confirmed due to the retrospective, non-randomized design of this study.

## 1 Introduction

Chronic obstructive pulmonary disease (COPD) is a common respiratory disease characterized by airway inflammation, mucus hypersecretion, and destruction of the lung parenchyma. The incidence is highest among middle-aged and elderly people. Studies have shown that the number of people suffering from this disease in China has reached up to 100 million, placing a heavy burden on society (1–3). Brennan et al. (4) reported that patients with COPD tend to present with clinical symptoms such as cough, sputum production, and wheezing. When in the acute exacerbation phase of chronic obstructive pulmonary disease (AECOPD), under the influence of bacterial and viral infections, air pollution, climate change and other factors, an accelerated decline in lung function, an increase in systemic inflammation, and a sharp aggravation of the condition can occur in a short period of time. This seriously affects the patient’s ability to work and even threatens their life and health. Yuan et al. (5) have found that, in addition to the clinical management of respiratory symptoms in AECOPD, the high incidence of digestive complications such as constipation has gradually attracted attention. Approximately 30–50% of patients with COPD experience constipation during an acute exacerbation. This is closely related to intestinal dysfunction and autonomic dysregulation caused by prolonged bed rest and side effects of medications. Constipation not only exacerbates the discomfort of abdominal distension and loss of appetite but also aggravates respiratory difficulties through diaphragm uplift, which will result in “respiratory-digestive” symptoms and even threaten life and health. The vicious cycle of “respiration-digestion” further prolongs hospitalization and increases the difficulty of treatment. Therefore, the adoption of scientific and effective clinical treatment is of great significance to these patients.

At present, clinical interventions for AECOPD with constipation mainly rely on Western symptomatic treatments, such as laxative drugs, gastrointestinal motility modifiers, and lifestyle adjustments. Still, these methods suffer from large individual differences in efficacy, obvious side effects (e.g., electrolyte disorders, drug dependence), and difficulties in fundamentally improving the interaction between intestinal function and systemic inflammatory state (6, 7). In recent years, traditional Chinese medicine (TCM) has demonstrated unique advantages in the intervention of AECOPD with constipation, and the advantages of TCM in multi-target regulation have been gradually emphasized, especially the combination of external treatment and internal therapies, which provides a new way of thinking for the management of the multi-system involvement of COPD. Indirect moxibustion, as an external therapy of Chinese medicine, stimulates acupoints through the heat generated by the burning of moxa fleece over a specific medium. This has the efficacy of warming the meridians and regulating qi and blood (8–10). Scholars such as Nakahara (11) have shown that indirect moxibustion, firstly documented in the “*Emergency Prescriptions Worth a Thousand Gold Pieces,*” which is “to use salt to be taken into the umbilicus, and indirect moxibustion for two or seven strokes on the upper part of the umbilicus,” can cure hernia, nourish the five flavors, pass urine and feces, with the help of indirect moxibustion’s warming efficacy of the drug ions through the skin, acupoints and meridians to the site of action, all the meridians and veins, with warming and tonifying the yuan–yang, strengthening the spleen and stomach, improving the role of significantly improving the lung function of patients with COPD, alleviating the inflammation of the airway, and through the regulation of the vagus nerve activity to promote gastrointestinal peristalsis. Meanwhile, Ma Ren Pill, as a classic intestine-moistening herbal formula, is composed of flaxseed, bitter almond, and rhubarb, which can lubricate the intestines, promote bowel movement, clear heat and stagnation, and is especially suitable for constipation of mixed excess-deficiency syndrome (12). Although both indirect moxibustion and Ma Ren Pill show potential value as monotherapy, the synergistic effect of combining the two in patients with AECOPD with constipation has not been fully investigated, and their mechanisms of action and clinical safety still need to be systematically verified.

Based on the above background, this study aims to evaluate the impact of indirect moxibustion combined with Ma Ren Pill on the time of resolution of constipation and the clinical symptoms of patients with chronic obstructive pulmonary disease in the acute exacerbation stage (AECOPD) who have constipation through a single-center retrospective analysis. The intervention mode of combining external and internal Chinese medicine treatments overcomes the limitations of the traditional Tongzhi method, which is prone to injuring positive qi. Ultimately, this study aims to promote standardization of the application of traditional Chinese medicine in respiratory system disease management.

## 2 Materials and methods

### 2.1 Statement of ethics

This study was approved by the Institutional Review Board and Ethics Committee of our hospital. Given that this study was retrospective and only de-identified patient data were used, informed consent was not required as there was no risk or adverse effect on patient care. This waiver complies with standard regulatory and ethical guidelines for retrospective studies.

### 2.2 Study design

This retrospective study included 102 patients with AECOPD and constipation who were treated between March 2023 and March 2024 at our hospital. They were divided into two groups based on the treatment modalities recorded in the electronic medical record system: 49 cases in the control group (treatment with Ma Ren Pill) and 53 cases in the observation group (indirect moxibustion with Ma Ren Pill).

### 2.3 Inclusion criteria

Inclusion criteria: (1) Western medical diagnosis of COPD meeting the relevant criteria in the *Clinical Guidelines for the Diagnosis and Treatment of Chronic Obstructive Pulmonary Disease* (13), classified as Grades II–III COPD; a TCM diagnosis conforming to standards in *Guidelines for the Diagnosis and Treatment of Chinese Medical Diseases in Traditional Chinese Medicine* (14), and the identification of phlegm-heat congestion of the lungs, with the primary symptoms of fever, cough, wheezing, chest tightness and chest pain, and viscous sputum, and the secondary symptoms of phlegm of yellowish consistency, irritability, thirst red face, yellow urine, dry stools, red tongue, yellow and greasy tongue coating, and a slippery pulse, which meets the identification criteria for heat accumulation and constipation; (2) age >18 years old; (3) availability of complete clinical data; and (4) not accompanied by serious neurological diseases.

Exclusion criteria: (1) severe liver or kidney dysfunction; (2) interstitial lung disease, poorly controlled diabetes mellitus (glycated hemoglobin ≥7.0% or presence of diabetic complications), or spontaneous pneumothorax; (3) allergy or allergic to the presence of the study drug; (4) patients with serious skin diseases; (5) combined with the heart, brain, liver, kidney and serious diseases; (6) suffering from other respiratory diseases such as tuberculosis, lung cancer.

### 2.4 Treatment

#### 2.4.1 Control group

Taking Ma Ren Pill (manufacturer: Hangzhou Hu Qing Yu Tang Pharmaceutical Co., Ltd., batch number: State Pharmaceutical License Z33020142), a classic TCM formula composed of 15 g of Semen Cannabis, 10 g of Semen Armeniacae Amarae, 6 g of Radix et Rhizoma Rhei, 12 g of Fructus Aurantii Immaturus (fried), 10 g of Cortex Magnoliae Officinalis (processed with ginger), and 12 g of Radix Paeoniae Alba (fried), formulated with refined honey as an excipient. The pills are water-honeyed, each 6 g, administered orally twice daily (morning and evening). Each 7-day period of continuous administration constituted one course. To achieve a total treatment duration of 2 months, the 7-day courses were repeated immediately and continuously without any interruption or washout between courses. Thus, each patient in the control group received approximately eight to nine repeated courses (60 days total, with 7 days per course, i.e., 8 full courses plus 4 days of the 9th course), ensuring consistent and uninterrupted medication throughout the observation period.

#### 2.4.2 Observation group

Based on the same Ma Ren Pill regimen as the control group, patients in the observation group additionally received indirect moxibustion. Fresh ginger was cut into slices with a diameter of 2–3 mm and a thickness of 2–5 mm, and several small holes were punctured in the center of each slice using a needle. Patients were assisted to remain in a supine position with the treatment sites fully exposed, after which the ginger slices were placed on the patients’ Shenque, Dazhong (KI4), Tianshu, Feishu (BL13), Mingmen (GV4), Tanzhong (CV17), Dingchuan (EX-B1), and Kongzui (LU6) acupoints. Lighting moxa cones for indirect moxibustion, moxa cone length 200 mm, diameter 18 mm. After lighting each acupuncture point, indirect moxibustion for 15 min. During this procedure, the patient needs to be instructed to pay close attention to the moxa burning situation; when the skin flushed, and the patient felt a clear sense of heat. Sessions were conducted 1 time per day. Each period of 7–10 consecutive days of daily indirect moxibustion constituted one course. To achieve a total treatment duration of 2 months, the 7–10-day course was repeated continuously without interruption between courses. Accordingly, each patient in the observation group received daily indirect moxibustion over the entire 2-month period, resulting in a total of 56 to 60 indirect moxibustion sessions (depending on whether a 7-day or 10-day course structure was completed). Precautions for indirect moxibustion: ① In the indirect moxibustion process, closely observe the changes in the condition. If the patient’s cough, sputum, wheezing and other symptoms worsen, or can not tolerate moxa smoke, indirect moxibustion should be stopped immediately; ② Observation of the patient’s skin, to prevent the ash off the skin, such as due to indirect moxibustion inadvertently scalded the skin, blisters appear locally, then release the water, and then coated with anti-inflammatory ointment, covered with antiseptic dressings, to prevent infection.

All patients (both groups) received continuous treatment for 2 months (60 days). For the control group, this consisted of uninterrupted repetition of the 7-day Ma Ren Pill courses (approximately 8–9 courses). For the observation group, this consisted of uninterrupted repetition of the 7–10-day indirect moxibustion courses (56–60 sessions in total) in addition to the same Ma Ren Pill regimen. It should be noted that the 60-day standardized full two-month treatment protocol is the unified complete intervention cycle for all enrolled patients, and hospitalization duration (approximately 11–13 inpatient days) only refers to the acute inpatient stay for initial AECOPD stabilization within this fixed 60-day window. After hospital discharge, all participants continued the remaining treatment via outpatient visits to complete the full 60-day treatment protocol. Relevant indices were collected on admission and on the second day after completing the full 60-day standardized treatment course. After discharge, patients in both groups continued the assigned treatment as outpatients: the control group continued oral Ma Ren Pill twice daily. In contrast, the observation group continued indirect moxibustion at our hospital’s Traditional Chinese Medicine outpatient department daily (returning to the hospital each day for supervised sessions). Adherence to the Ma Ren Pill regimen was assessed through pill counts at each outpatient visit (every 7 days), and adherence to indirect moxibustion was ensured through direct observation during each session, with attendance records maintained for all participants. Patients who missed more than three consecutive sessions or <80% of scheduled treatments were to be excluded from the per-protocol analysis, though in practice no patient met these exclusion thresholds.

### 2.5 General data collection

Collected through the medical record system records, including age, sex (male/female), duration of AECOPD, severity of disease (mild/moderate), duration of bloating, smoking history (number of cigarettes smoked per day > 1, and the duration of continuous time > 1 year or the time of quitting < 1 year), and history of alcohol consumption (amount of alcohol consumed per day > 1 drinking volume, and the duration of continuous time > 1 year or the time of quitting < 1 year (1 drinking volume is 45 mL liquor/360 mL beer/120 mL fruit wine), baseline bowel movement frequency (week), and common comorbidities (hypertension, diabetes, coronary heart disease). In addition, to address potential confounding by indication, we also collected data on standard Western medical treatments for AECOPD administered during hospitalization, including systemic or inhaled glucocorticoids, antibiotics, and bronchodilators (short-acting *β*₂-agonists and/or anticholinergics). These variables were compared between the two groups to ensure that any observed differences in outcomes were not simply due to imbalances in concomitant AECOPD management.

### 2.6 Primary outcome indicator

The primary outcome indicator includes the constipation disappearance time, which was recorded from the electronic medical record system. Constipation disappearance time was prespecified as the primary outcome before data extraction and statistical analysis. Constipation disappearance time was defined as the number of days from treatment initiation to the first documented normal bowel movement (Bristol Stool Form Scale types 3–4) without the use of rescue laxatives.

### 2.7 Secondary outcome indicators

(1) Constipation symptom score, respiratory symptoms score, and hospitalization duration

① Constipation symptom score: This score was assessed using a modified version of a previously validated constipation symptom scale originally developed for functional constipation. The original scale has demonstrated good internal consistency (Cronbach’s *α* = 0.87) and test–retest reliability (intraclass correlation coefficient, ICC = 0.82). This scale includes six dimensions that are particularly relevant to AECOPD patients with constipation: (a) stool characteristics (hard, lumpy, dry vs. soft, formed); (b) defecation effort (need for straining during bowel movements); (c) defecation time (minutes required for each bowel movement); (d) sensation of incomplete evacuation (feeling of incomplete emptying after defecation); (e) abdominal bloating/distension; and (f) defecation frequency (bowel movements per week). Each dimension was graded on a 4-point scale from 0 to 3 (0 = none/normal, 1 = mild, 2 = moderate, 3 = severe). The total score ranged from 0 to 18 points, with higher scores indicating more severe constipation symptoms. Assessments were performed at baseline (before treatment) and on the second day following completion of the 2-month treatment period. ② Respiratory symptoms score: This score evaluated six respiratory symptom dimensions: (a) cough frequency and intensity; (b) sputum production (amount and characteristics); (c) shortness of breath (dyspnea on exertion or at rest); (d) wheezing; (e) sputum volume; and (f) lung rales (auscultation findings). These items were derived from a combination of the COPD Assessment Test (CAT) and the modified Medical Research Council (mMRC) dyspnea scale, both of which are well-validated in COPD populations. Each dimension was graded on a 4-point scale from 0 to 3 (0 = none, 1 = mild, 2 = moderate, 3 = severe). The total score ranged from 0 to 18 points, with higher scores indicating more severe respiratory symptoms. All symptom scores were assessed by trained physicians who were not involved in the patients’ clinical care and were unaware of the study’s grouping or hypothesis; however, given the retrospective nature of this study, these physicians were not formally blinded to treatment allocation in the prospective trial sense, as group assignment was determined by historical treatment records rather than randomized allocation. ③ Hospitalization duration: This metric was extracted from electronic medical records. Hospitalization duration is defined as the number of consecutive inpatient days from admission for AECOPD and constipation to discharge, and all inpatient stays occurred within the unified 60-day treatment protocol. A shorter hospitalization duration indicated faster relief of acute symptoms with the assigned intervention, while all patients completed the remaining outpatient treatment days to fulfill the full 60-day intervention cycle.

(2) Lung function

The patient’s first second expiratory volume with exertion (FEV_1_), peak expiratory flow rate (PEF), and forced vital capacity (FVC) were measured using a pulmonary function tester (Yeager, Germany, MS-Diffusion model), and FEV_1_/FVC (the ratio of first second expiratory volume with exertion to forced vital capacity) was calculated.

(3) Blood gas indicators and inflammatory factors

Arterial blood samples were obtained via radial artery puncture. For the measurement of partial pressure of oxygen (PO₂) and partial pressure of carbon dioxide (PCO₂) using a blood gas analyzer (Shanghai Ouqi Electronic Technology Co., Ltd.). For the assessment of inflammatory markers, 5 mL of fasting venous blood was collected from patients in the morning, centrifuged at 3000 r/min for 10 min (centrifuge model: LL900, manufacturer: Luoyang Hongshi Machinery Equipment Co., Ltd.), and the serum was separated. Tumor necrosis factor alpha (TNF-*α*), interleukin-8 (IL-8), and C-reactive protein (CRP) were measured by enzyme-linked immunosorbent assay, with kits purchased from Nanjing Jiancheng Bioengineering Institute.

(4) Clinical efficacy

The assessment was conducted in accordance with the “Guidelines for Diagnosis and Treatment of Common Diseases in Internal Medicine of Traditional Chinese Medicine.” The specific criteria were as follows:

①Cured: Normal bowel movements without the need for laxatives, with stool consistency becoming moist and well-formed, defecation occurring once every 1–2 days, and no recurrence of constipation within 1 week after treatment completion. All other symptoms (cough, sputum, wheezing, etc.) completely disappeared. ② Obvious effect: Bowel movements occur once every 3 days, stool consistency is soft, and other symptoms (cough, sputum, and dyspnea) basically disappeared or markedly improved. ③Effective: Bowel movements occur once within 3 days, with a soft stool but stool remains hard or dry; other symptoms show partial improvement. ④ Ineffective: No bowel movement occurs within 1 week, or laxative assistance is still required to achieve defecation, and stool remains dry and lumpy; respiratory symptoms show no significant improvement. Total effective rate = (number of cured + number of remarkable effect + number of effective)/total number of cases×100%.

(5) Quality of life

The Generic Quality of Life Inventory-74 (GQOLI-74) (15) was used to assess patients’ quality of life before and after treatment. The questionnaire included four dimensions of material life, somatic function, social function, and psychological function. Each dimension is scored on a scale of 0 to 100, with higher scores indicating better quality of life.

(6) Adverse reaction situation

Adverse events related to the interventions were retrospectively collected from electronic medical records throughout the 2-month treatment period. For the control group (Ma Ren Pill alone), we recorded any gastrointestinal symptoms, including nausea, vomiting, diarrhea, and abdominal pain, as well as any signs of electrolyte disturbance or drug dependence. For the observation group (indirect moxibustion plus Ma Ren Pill), in addition to the above, we specifically monitored for adverse local skin conditions at the moxibustion sites (Shenque, Dazhong, Tianshu, Lung Yu, Mingmen, Tanzhong, Dingchuan, and Kongzui points), including skin erythema, blisters, burns, pain, and infection. We also recorded any patient-reported intolerance to moxa smoke (e.g., cough, throat irritation, and dyspnea). All adverse events were graded according to the Common Terminology Criteria for Adverse Events (CTCAE) version 5.0. The incidence of adverse events was compared between the two groups using the chi-square test or Fisher’s exact test, as appropriate.

### 2.8 Statistical treatment

Statistical analysis was performed using IBM SPSS Statistics version 27.0 (IBM Corp., Armonk, NY, USA). Continuous variables were presented as mean ± standard deviation (SD). For comparisons between the two groups, independent-samples *t*-tests were used for normally distributed continuous variables, and Mann–Whitney U tests were used for non-normally distributed variables. For within-group comparisons before and after treatment, paired t-tests were used for normally distributed data, and Wilcoxon signed-rank tests were used for non-normally distributed data. Categorical variables were compared using the chi-square test or Fisher’s exact test, as appropriate. Given the multiple outcome comparisons (including constipation symptoms, pulmonary function, blood gas indices, inflammatory factors, quality of life, and adverse events), this study applied the Benjamin–Hochberg false discovery rate (FDR) correction to control for type I error. Adjusted *p* values are reported alongside the original p values. In addition, to facilitate clinical interpretation, the study calculated Cohen’s *d* for independent-samples *t*-tests as a standardized effect size, which allows comparison across different outcome measures. For categorical outcomes, the study presents odds ratios (ORs) with 95% confidence intervals (CIs). All effect sizes are reported alongside the corresponding original and FDR-adjusted *p* values. Statistical significance was defined as a two-sided FDR-adjusted *p* < 0.05 (unless otherwise specified).

## 3 Results

### 3.1 Comparison of clinical baseline data between the two groups of patients

Comparison of the baseline data of age, sex, duration of AECOPD, severity of disease, duration of abdominal distension, history of smoking, history of alcohol consumption, baseline bowel movement frequency, hypertension, diabetes, coronary heart disease, as well as the use of glucocorticoids, antibiotics, and bronchodilators between the two groups showed no statistically significant differences (*p* > 0.05), suggesting that the groups are comparable, see Table 1.

**Table 1:** Comparison of clinical baseline data between the two groups of patients.

Indicators	Observation group (*n* = 53)	Control group (*n* = 49)	Cohen’s *d*/OR (95% CI)	*p*-value	Adjusted *p*-value
Age (year)	54.79 ± 6.53	54.52 ± 6.61	Cohen’s *d* = 0.04 (−0.35 ~ 0.43)	0.836	0.836
Sex (*n*, %)			OR = 1.25 (0.58 ~ 2.71)	0.572	0.572
Male	30 (56.60)	25 (51.02)			
Female	23 (43.40)	24 (48.98)			
AECOPD course (*d*)	3.78 ± 0.32	3.69 ± 0.40	Cohen’s *d* = 0.25 (−0.14 ~ 0.64)	0.211	0.211
Severity of illness (*n*, %)			OR = 0.97 (0.46 ~ 2.08)	0.943	0.943
Mild	31 (58.49)	29 (59.18)			
Moderate	22 (41.51)	20 (40.82)			
Abdominal distension course (*d*)	2.59 ± 0.21	2.53 ± 0.26	Cohen’s *d* = 0.25 (−0.14 ~ 0.65)	0.201	0.201
Smoking history (*n*, %)			OR = 0.25 (−0.14 ~ 0.65)	0.718	0.718
Yes	18 (33.96)	15 (30.61)			
No	35 (66.04)	34 (69.39)			
Drinking history (*n*, %)			OR = 1.40 (0.49 ~ 4.01)	0.535	0.535
Yes	10 (18.87)	7 (14.29)			
No	43 (81.13)	42 (85.71)			
Baseline bowel movement frequency (week)	2.34 ± 0.56	2.41 ± 0.62	Cohen’s *d* = −0.12 (−0.51 ~ 0.27)	0.551	0.551
Hypertension (*n*, %)			OR = 1.14 (0.45 ~ 2.90)	0.784	0.784
Yes	12 (22.64)	10 (20.41)			
No	41 (77.36)	39(79.59)			
Diabetes (*n*, %)			OR = 1.09 (0.34 ~ 3.50)	0.884	0.884
Yes	7 (13.21)	6 (12.24)			
No	46(86.79)	43(87.76)			
Coronary heart disease (*n*, %)			OR = 0.92 (0.25 ~ 3.36)	0.896	0.896
Yes	5 (9.43)	5 (10.20)			
No	48(90.57)	44 (89.80)			
Glucocorticoid use (*n*, %)			OR = 1.10 (0.42 ~ 2.90)	0.845	0.845
Yes	43 (81.13)	39 (79.59)			
No	10(18.87)	10(20.41)			
Antibiotic use (*n*, %)			OR = 1.09 (0.33 ~ 3.64)	0.885	0.885
Yes	47 (88.68)	43 (87.76)			
No	6(11.32)	6(12.24)			
Bronchodilator use (*n*, %)			OR = 1.48 (0.31 ~ 6.98)	0.617	0.617
Yes	50 (94.34)	45 (91.84)			
No	3(5.66)	4 (8.16)			

OR is odds ratio; CI is confidence interval.

### 3.2 Comparison of constipation disappearance time

**Figure 1:**
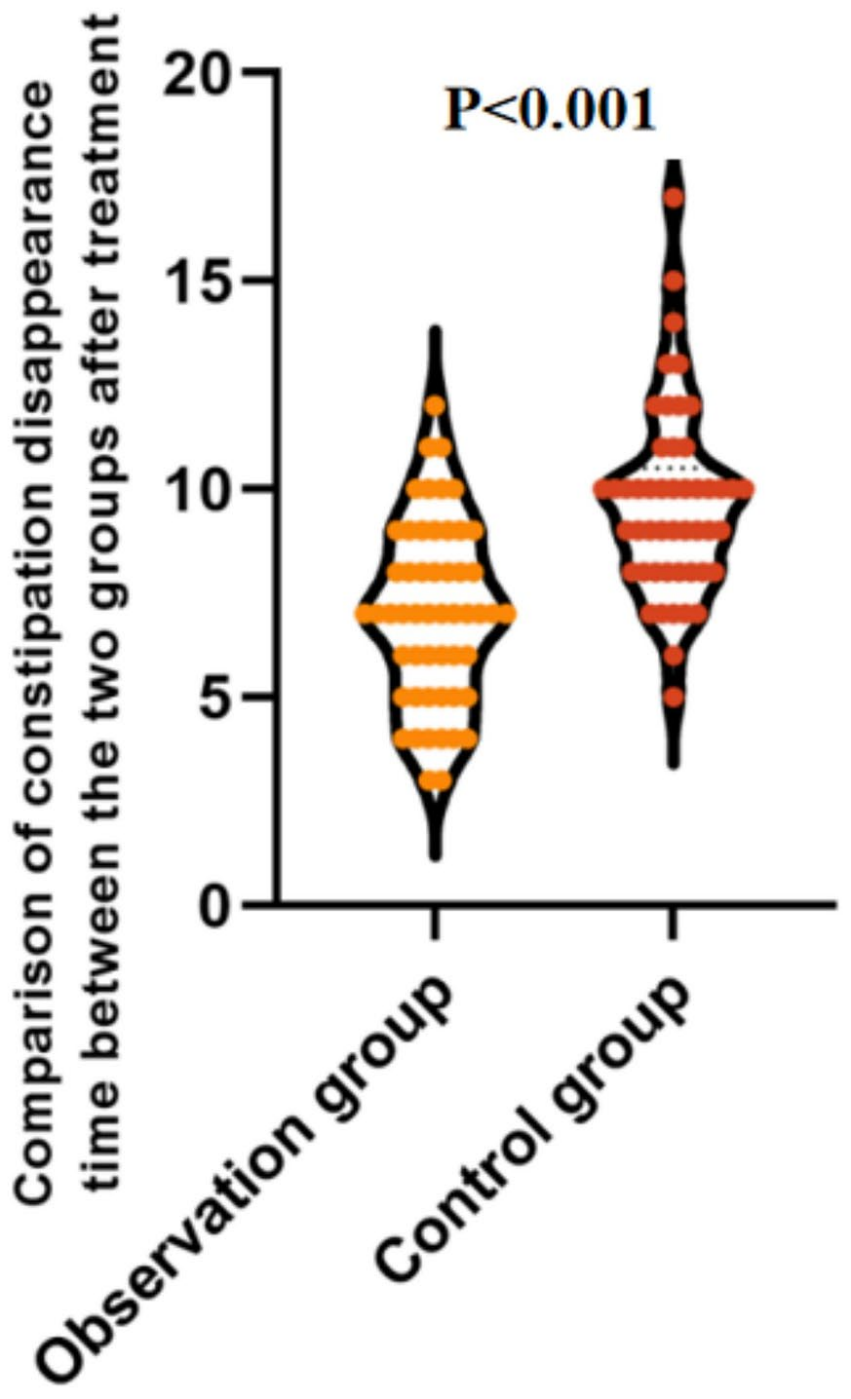
Comparison of constipation disappearance time.

After treatment, the constipation resolution time was significantly shorter in the observation group (7.05 ± 2.19 vs. 9.67 ± 2.31 days) than in the control group, and the difference was statistically significant (adjusted *p* < 0.05). These findings indicate that the treatment with indirect moxibustion combined Ma Ren Pill was associated with a shorter constipation resolution time in patients with constipation in AECOPD (Figure 1).

### 3.3 Comparison of constipation symptom score, respiratory symptoms score, and hospitalization duration

Constipation and respiratory symptom scores are important indicators for evaluating the clinical symptoms of patients with AECOPD with constipation, and hospitalization duration is a reflection of the clinical recovery effect of patients. Before treatment, there was no statistically significant difference between the constipation and respiratory symptom scores of patients in the observation group and the control group (adjusted *p* > 0.05) (Table 2).

**Table 2:** Comparison of constipation symptom score, respiratory symptoms score, and hospitalization duration ( $\bar{x}\pm s$ ).

Indicators	Observation group (*n* = 53)	Control group (*n* = 49)	Cohen’s *d* (95% CI)	*p-*value	Adjusted *p-*value
Constipation symptom score (score)					
Before treatment	13.83 ± 1.49	13.47 ± 1.52	0.24 (−0.15 ~ 0.63)	0.230	0.230
After treatment	3.25 ± 1.37^*^	5.95 ± 1.50^*^	−1.87 (−2.33 ~ −1.40)	<0.001	<0.001
Respiratory symptom score (score)					
Before treatment	14.36 ± 1.57	14.17 ± 1.62	0.12 (−0.27 ~ 0.51)	0.549	0.549
After treatment	3.19 ± 1.26^*^	5.81 ± 1.49^*^	−1.89 (−2.36 ~ −1.42)	<0.001	<0.001
hospitalization duration (d)	11.36 ± 1.29	13.53 ± 1.48	−1.57 (−2.02 ~ −1.12)	<0.001	<0.001

CI is confidence interval; * indicates that the p-value of the paired t-test is less than 0.05.

**Figure 2:**
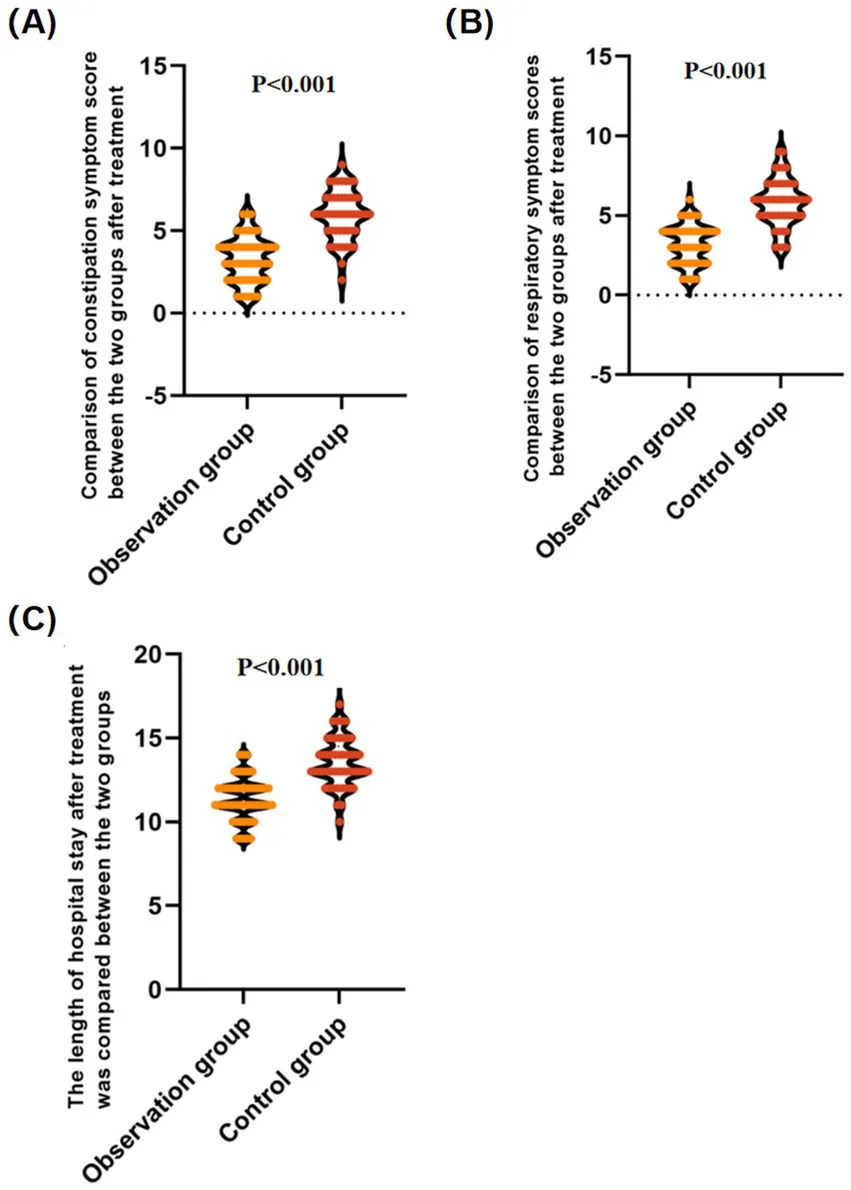
Comparison of scores of constipation, respiratory system symptoms, and the recovery situation. **(A)** Comparison of constipation symptom score between the two groups after treatment; **(B)** Comparison of respiratory symptom scores between the two groups after treatment; **(C)** The length of hospital stay after treatment.

After treatment, the constipation symptom score (3.25 ± 1.37 vs. 5.95 ± 1.50 points), respiratory symptom score (3.19 ± 1.26vs.5.81 ± 1.49 points), and hospitalization duration (11.36 ± 1.29 vs. 13.53 ± 1.48 days) were all lower than those of the control group and the difference was statistically significant (adjusted *p* < 0.05), thus indicating that the treatment of indirect moxibustion and Ma Ren Pill was associated with greater improvement on constipation and respiratory symptoms in patients with constipation in AECOPD, with constipation and the improvement was higher in the combined treatment (Figure 2).

### 3.4 Comparison of pulmonary function indices between the two groups of patients before and after treatment

PEF reflects the degree of airway patency, FEV_1_ reflects the volume of expiratory effort, and FEV_1_/FVC reflects the degree of airway obstruction, all of which are the core indicators in lung function testing. Before treatment, the comparison of PEF, FEV_1,_ and FEV_1_/FVC between patients in the observation group and the control group showed that the difference was not statistically significant (adjusted *p* > 0.05) (Table 3).

**Table 3:** Comparison of pulmonary function indices between the two groups of patients before and after treatment (*x̄* ± s).

Indicators	Observation group (*n* = 53)	Control group (*n* = 49)	Cohen’s *d* (95% CI)	*p*-value	Adjusted *p*-value
FEV₁ (L)					
Before treatment	1.84 ± 0.70	1.82 ± 0.69	0.03 (−0.36 ~ 0.42)	0.885	0.885
After treatment	2.91 ± 1.06^*^	2.25 ± 0.81^*^	0.69 (0.28 ~ 1.10)	0.001	0.003
PEF (L/s)					
Before treatment	4.31 ± 0.82	4.30 ± 0.80	0.01 (−0.38 ~ 0.40)	0.950	0.950
After treatment	5.40 ± 0.95^*^	4.91 ± 0.86^*^	0.54 (0.13 ~ 0.94)	0.008	0.012
FEV₁/FVC (%)					
Before treatment	58.84 ± 7.36	58.82 ± 7.35	0.00 (−0.39 ~ 0.39)	0.989	0.989
After treatment	71.18 ± 8.55^*^	66.28 ± 8.04^*^	0.59 (0.18 ~ 1.00)	0.004	0.008

FEV_1_ is expiratory volume at exertion in the first second, PEF is peak expiratory flow rate, FVC is expiratory lung volume, and FEV_1_/FVC is the ratio of expiratory volume at exertion in the first second to expiratory lung volume; CI is confidence interval; * indicates that the *p*-value of the paired *t*-test is less than 0.05.

**Figure 3:**
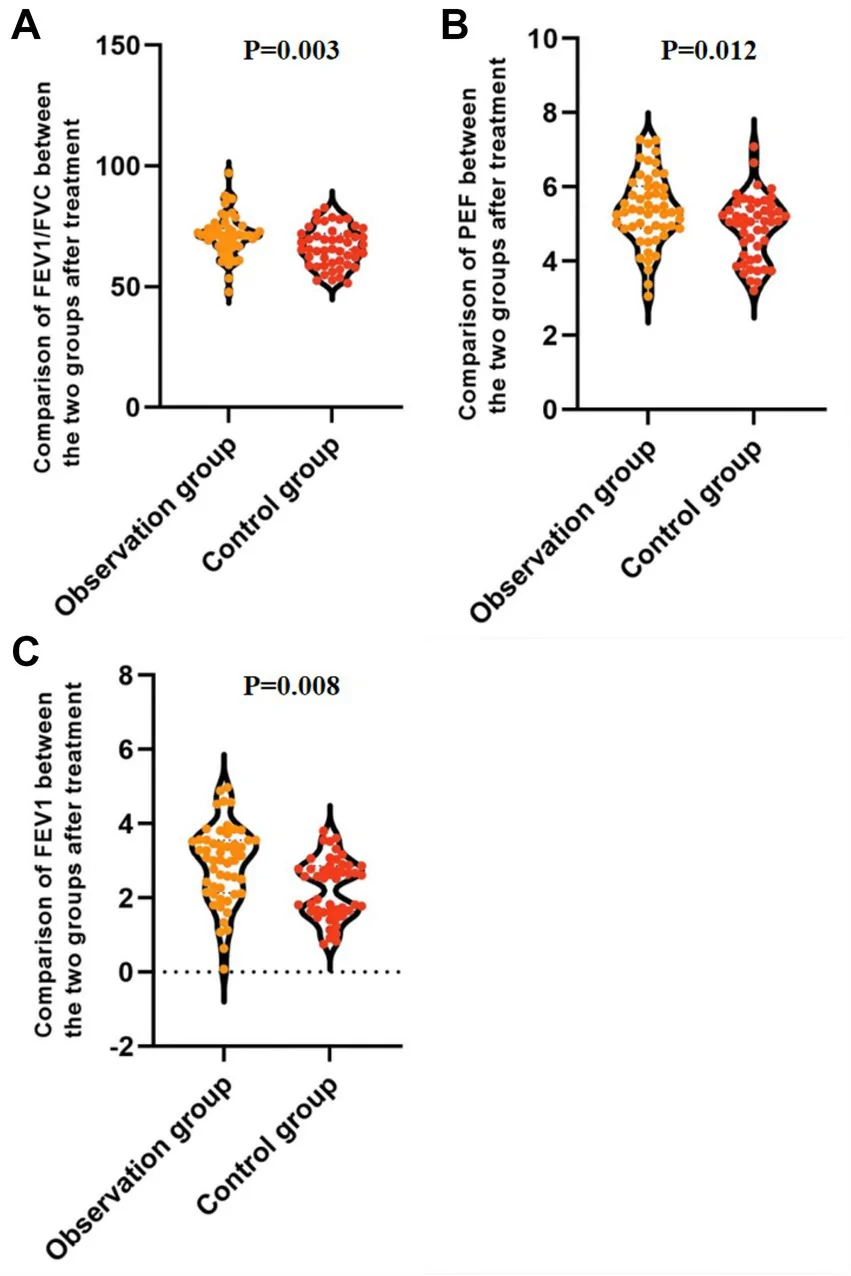
Comparison of pulmonary function indices in two groups of patients. FEV_1_/FVC is the ratio of expiratory volume in the first second to expiratory lung volume; PEF is peak expiratory flow rate; FEV_1_ is expiratory volume in the first second. **(A)** Comparison of FEV_1_/FVC between the two groups after treatment; **(B)** Comparison of PEF between the two groups after treatment; **(C)** Comparison of FEV_1_ between the two groups after treatment.

After treatment, PEF (5.40 ± 0.95 vs. 4.91 ± 0.86 L/s), FEV_1_ (2.91 ± 1.06 vs. 2.25 ± 0.81 L), and FEV_1_/FVC (71.18 ± 8.55 vs. 66.28 ± 8.04%) of the patients in the Observation Group were higher than those of the control group, and the difference was statistically significant (adjusted *p* < 0.05). This finding suggests an association between the combined intervention regimen and improved pulmonary function parameters, but causal therapeutic improvement cannot be inferred from this retrospective non-randomized dataset (Figure 3).

### 3.5 Comparison of blood gas indices and inflammatory factor indices between the two groups of patients before and after treatment

PO_2_ and PCO_2_ reflect the oxygen-exchange efficiency of the lungs, the state of tissue oxygenation, and the respiratory status of the tissues and are core indicators for assessing the external respiratory function; TNF-*α*, IL-8, and CRP are important inflammatory markers, reflecting the inflammatory response of the body. Before treatment, the comparison of PO_2_, PCO_2_, TNF-α, IL-8, and CRP between AECOPD patients with constipation and patients of the observation group and the control group showed that the difference was not statistically significant (adjusted *p* > 0.05) (Table 4).

**Table 4:** Comparison of blood gas indices and inflammatory factor indices between the two groups of patients before and after treatment (*x̄* ± s).

Indicators	Observation group (*n* = 53)	Control group (*n* = 49)	Cohen’s *d* (95% CI)	P value	Adjusted P value
PO₂ (mmHg)					
Before treatment	48.60 ± 5.23	48.65 ± 5.22	−0.01 (−0.40 ~ 0.38)	0.962	0.962
After treatment	84.68 ± 7.60^*^	78.49 ± 7.35^*^	0.83 (0.42 ~ 1.24)	<0.001	<0.001
PCO₂ (mmHg)					
Before treatment	69.75 ± 4.80	69.77 ± 4.84	−0.00 (−0.39 ~ 0.39)	0.983	0.983
After treatment	45.64 ± 3.53^*^	50.46 ± 3.33^*^	−1.40 (−1.85 ~ −0.95)	<0.001	<0.001
TNF-α (ng/L)					
Before treatment	68.94 ± 11.88	69.33 ± 12.31	−0.03 (−0.42 ~ 0.36)	0.871	0.871
After treatment	38.24 ± 8.25^*^	47.45 ± 10.56^*^	−0.96 (−1.38 ~ −0.54)	<0.001	<0.001
IL-8 (ng/L)					
Before treatment	63.38 ± 8.93	62.47 ± 9.61	0.10 (−0.29 ~ 0.49)	0.621	0.621
After treatment	40.68 ± 6.19^*^	47.21 ± 5.30^*^	−1.13 (−1.56 ~ −0.70)	<0.001	<0.001
CRP (mg/L)					
Before treatment	68.09 ± 17.76	67.47 ± 18.38	0.03 (−0.36 ~ 0.42)	0.863	0.863
After treatment	9.66 ± 1.38^*^	13.31 ± 2.97^*^	−1.57 (−2.02 ~ −1.12)	<0.001	<0.001

PO_2_ is partial pressure of blood oxygen, PCO_2_ is partial pressure of arterial blood carbon dioxide, TNF-α is tumor necrosis factor α, IL-8 is interleukin-8, and CRP is C-reactive protein; CI is confidence interval; * indicates that the *p*-value of the paired *t*-test is less than 0.05.

**Figure 4:**
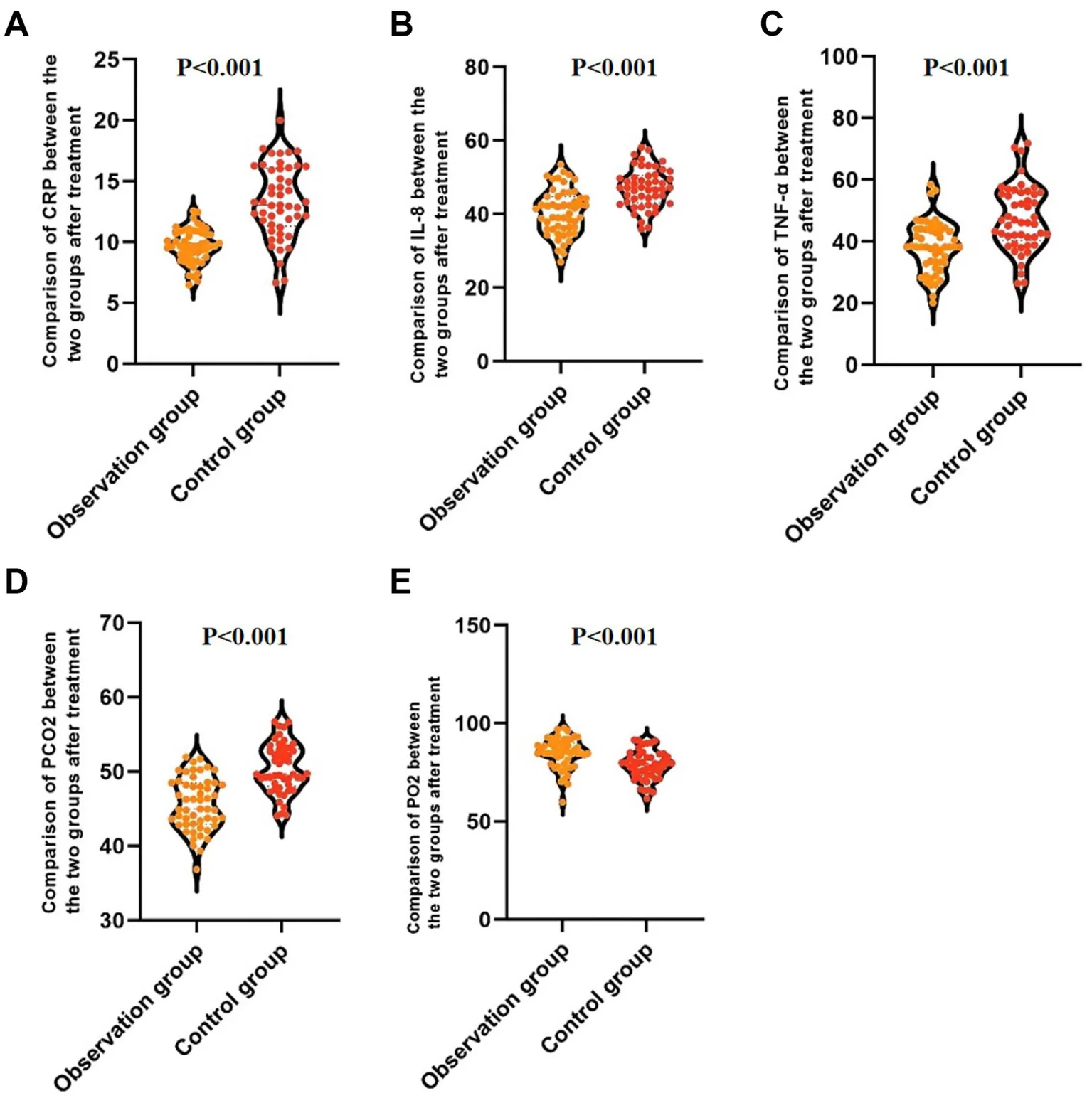
Comparison of blood gas indices and inflammatory factor indices between two groups of patients. CRP is C-reactive protein, IL-8 is interleukin-8, TNF-*α* is tumor necrosis factor α, PCO_2_ is partial pressure of arterial blood carbon dioxide, and PO_2_ is partial pressure of blood oxygen. **(A)** Comparison of CRP between the two groups after treatment; **(B)** Comparison of IL-8 between the two groups after treatment; **(C)** Comparison of TNF-*α* between the two groups after treatment; **(D)** Comparison of PCO_2_ between the two groups after treatment; **(E)** Comparison of PO_2_ between the two groups after treatment.

After treatment, PO_2_ (84.68 ± 7.60 vs. 78.49 ± 7.35 mmHg) was significantly higher in AECOPD with constipation patients in the observation group than in the control group, while levels of PCO_2_ (45.64 ± 3.53 vs. 50.46 ± 3.33 mmHg), TNF-*α* (38.24 ± 8.25 vs. 47.45 ± 10.56 ng/L), IL-8 (40.68 ± 6.19 vs.47.21 ± 5.30 ng/L), and CRP (9.66 ± 1.38 vs.13.31 ± 2.97 mg/L) were correspondingly reduced relative to the control group (adjusted *p* < 0.05). These parallel changes indicate an observational association between the combined moxibustion-Ma Ren Pill treatment and improved blood gas exchange and attenuated systemic inflammation, without confirming direct causal anti-inflammatory or oxygenation-enhancing effects (see Figure 4).

### 3.6 Comparison of clinical efficacy between the two groups of patients

The total effective rate is used to assess the efficacy of the treatment program, and the higher the total effective rate, the higher the treatment effect. The total effective rate was significantly higher in the observation group (98.11%) relative to the control group (83.67%), with OR = 10.15, 95% CI = (1.22–84.34), adjusted *p* = 0.015. This outcome demonstrates an association between the combined intervention and superior composite clinical response, yet this subjective physician-assessed endpoint must be interpreted cautiously given the study’s retrospective non-randomized design (see Table 5).

**Table 5:** Comparison of clinical efficacy between the two groups of patients (*n*, %).

Index	Observation group (*n* = 53)	Control group (*n* = 49)	OR (95% CI)	*p*-value	Adjusted *p*-value
Recovery	33 (62.26)	20 (40.82)		–	
Remarkable	13 (24.53)	14 (28.57)		–	
Effective	6 (11.32)	7 (14.29)		–	
Ineffective	1 (1.89)	8 (16.33)		–	
Total effective	52 (98.11)	41 (83.67)	10.15 (1.22 ~ 84.34)	0.010	0.015

OR is odds ratio; CI is confidence interval.

### 3.7 Comparison of the quality of life between the two groups of patients before and after treatment

Quality of life is a multidimensional concept, often used as an important indicator for assessing the effectiveness of policies. Before treatment, the comparison of material life, somatic function, social function, and psychological function scores of the observation group and control group showed no statistically significant differences (*p* > 0.05) (see Table 6).

**Table 6:** Comparison of the quality of life between the two groups of patients before and after treatment (points, *x̄* ± s).

Indicators	Observation group (*n* = 53)	Control group (*n* = 49)	Cohen’s *d* (95% CI)	*p*-value	Adjusted *p*-value
Material life					
Before treatment	60.23 ± 6.61	60.28 ± 6.58	−0.01 (−0.40 ~ 0.38)	0.970	0.970
After treatment	75.42 ± 7.92*	70.21 ± 7.65*	0.67 (0.26 ~ 1.08)	<0.001	0.001
Somatic function					
Before treatment	55.21 ± 6.48	55.59 ± 6.52	−0.06 (−0.45 ~ 0.33)	0.769	0.769
After treatment	75.05 ± 7.62*	70.53 ± 7.18*	0.61 (0.20 ~ 1.02)	0.002	0.004
Social function					
Before treatment	58.37 ± 6.21	58.79 ± 6.20	−0.07 (−0.46 ~ 0.32)	0.733	0.733
After treatment	74.12 ± 7.61*	70.52 ± 7.12*	0.49 (0.08 ~ 0.89)	0.014	0.020
Psychological function					
Before treatment	54.31 ± 6.48	54.35 ± 6.51	−0.01 (−0.40 ~ 0.38)	0.975	0.975
After treatment	70.25 ± 7.95*	65.21 ± 7.11*	0.66 (0.25 ~ 1.07)	0.001	0.003

CI is confidence interval; * indicates that the *p*-value of the paired *t*-test is less than 0.05.

**Figure 5:**
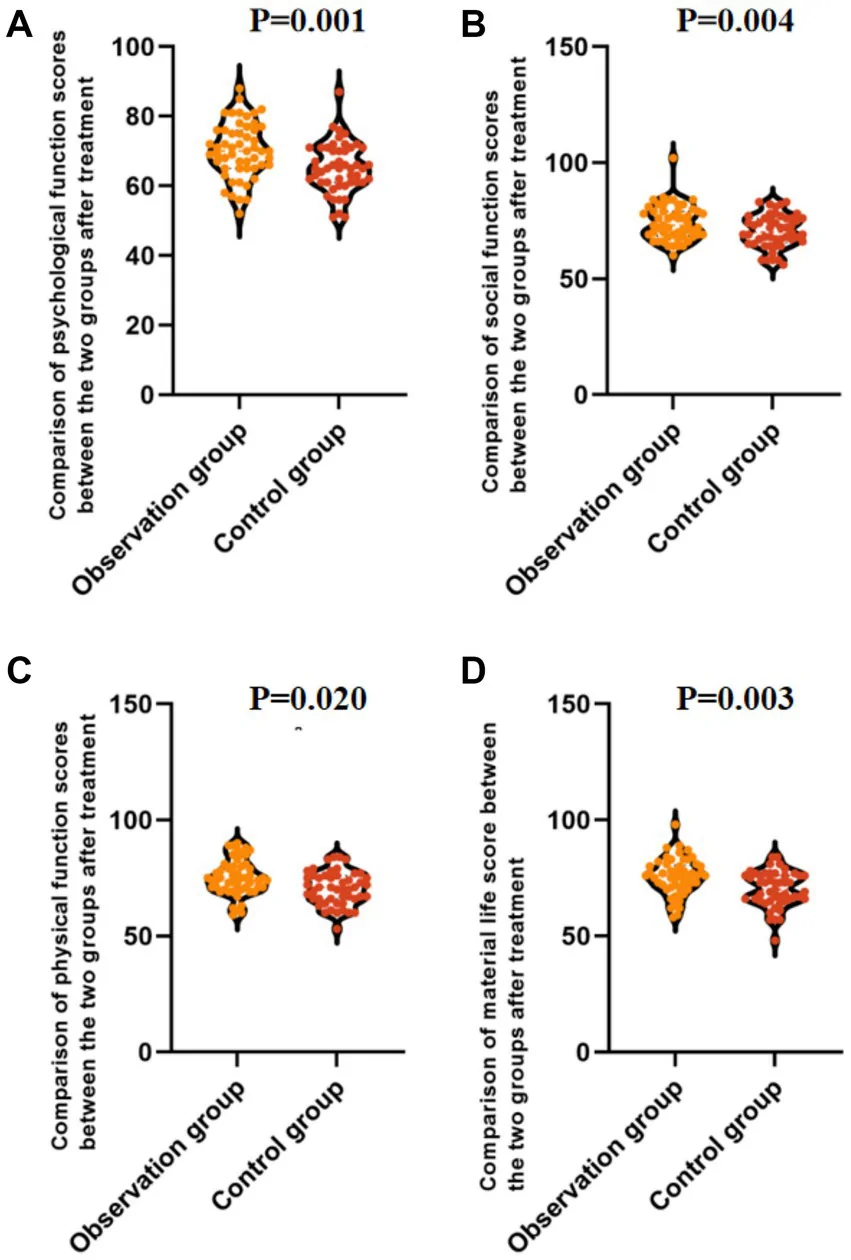
Comparison of quality of life after treatment of AECOPD with constipation between two groups. **(A)** Comparison of psychological function scores between the two groups after treatment; **(B)** Comparison of social function scores between the two groups after treatment; **(C)** Comparison of physical function scores between the two groups after treatment; **(D)** Comparison of material life score between the two groups after treatment.

After treatment, the observation group patients’ material life (75.42 ± 7.92 vs. 70.21 ± 7.65 scores), somatic function (75.05 ± 7.62 vs. 70.53 ± 7.18 scores), social function (74.12 ± 7.61 vs. 70.52 ± 7.12) scores), and psychological function (70.25 ± 7.95 vs. 65.21 ± 7.11 points) scores were higher than those of the control group, and the difference was statistically significant (*p* < 0.05). This observation supports a correlative relationship between combined indirect moxibustion and Ma Ren Pill regimen and improved quality-of-life scores, rather than proving the intervention directly improves patient quality of life (Figure 5).

### 3.8 Comparison of adverse reaction situation between the two groups

Adverse events related to the interventions were monitored and recorded throughout the 2-month treatment period. As shown in Table 7, no serious adverse events (grade ≥3) occurred in either group. In the control group, 2 patients (4.08%) reported mild nausea. In the observation group, 3 patients (5.66%) developed local skin erythema, and 1 patient (1.89%) reported moxa smoke intolerance. No blisters, burns, diarrhea, or electrolyte disturbances were observed. The overall incidence of adverse events was comparable between the two groups (7.55% in the observation group vs. 4.08% in the control group; OR = 1.92, 95% CI = (0.33–11.02), adjusted *p* = 0.457).

**Table 7:** Comparison of adverse events between the two groups (*n*, %).

Adverse event	Observation group (*n* = 53)	Control group (*n* = 49)	OR (95% CI)	*p*	Adjusted *p*-value
Nausea	0 (0.00)	2 (4.08)			
Local skin erythema	3 (5.66)	0 (0.00)			
Moxa smoke intolerance (cough/throat irritation)	1 (1.89)	0 (0.00)			
Blisters	0 (0.00)	0 (0.00)			
Skin burns	0 (0.00)	0 (0.00)			
Diarrhea	0 (0.00)	0 (0.00)			
Electrolyte disturbance	0 (0.00)	0 (0.00)			
Total adverse events	4 (7.55)	2 (4.08)	1.92 (0.33 ~ 11.02)	0.457	0.457

OR is odds ratio; CI is confidence interval.

## 4 Discussion

AECOPD corresponds to “lung distension” in traditional Chinese medicine (TCM), with core pathogenesis involving chronic lung deficiency, phlegm retention, and failure of lung qi descent, which are often exacerbated by external pathogenic invasion. Constipation frequently co-occurs in AECOPD, driven by reduced mobility, diaphragmatic dysfunction, medication side effects, and autonomic dysregulation, which may further elevate abdominal pressure, restrict respiratory mechanics, and amplify systemic inflammation, forming a gut–lung vicious cycle (16–18). Current standard Western care for AECOPD includes bronchodilators, glucocorticoids, antibiotics, and oxygen therapy, while constipation is typically managed with osmotic laxatives and lifestyle modification (13, 14). However, laxatives primarily provide symptomatic relief with limited effects on pulmonary function or systemic inflammation, and they carry risks of electrolyte disturbance and drug dependence (6, 7, 19–21). TCM offers multi-target regulation for complex comorbidities: Ma Ren Pill is a classic formula for mixed-deficiency-excess constipation, while indirect moxibustion is an external TCM that is traditionally used to warm meridians and regulate qi and blood (19). Prior evidence on their combined use for AECOPD with constipation remains limited (31).

The most compelling findings of this study derive from the objective physiological measurements. Specifically, the combined therapy was associated with a mean increase in FEV₁ of 0.66 L (from 2.25 ± 0.81 L to 2.91 ± 1.06 L) and in PEF of 0.49 L/s (from 4.91 ± 0.86 L/s to 5.40 ± 0.95 L/s) than Ma Ren Pill alone, with corresponding improvements in FEV₁/FVC ratio (from 66.28 ± 8.04% to 71.18 ± 8.55%). These changes in pulmonary function parameters are clinically meaningful, as they exceed the minimal clinically important difference (MCID) of 0.10–0.14 L for FEV₁ and 0.25 L/s for PEF established in COPD populations, suggesting that the addition of indirect moxibustion may confer tangible benefits in airway patency and ventilatory capacity. Furthermore, the combined intervention was associated with a marked improvement in blood gas exchange, as evidenced by a mean increase in PO₂ of 6.19 mmHg (from 78.49 ± 7.35 mmHg to 84.68 ± 7.60 mmHg) and a mean reduction in PCO₂ of 4.82 mmHg (from 50.46 ± 3.33 mmHg to 45.64 ± 3.53 mmHg). These changes reflect enhanced oxygen uptake and carbon dioxide clearance, which are critical endpoints in AECOPD management and correlate with reduced respiratory work and improved tissue oxygenation. Equally notable are the changes in systemic inflammatory markers: the combined regimen was associated with a mean reduction of 9.21 ng/L in TNF-*α*, 6.53 ng/L in IL-8, and 3.65 mg/L in CRP relative to the control group. Given that elevated levels of these pro-inflammatory cytokines are independently associated with worse clinical outcomes and increased mortality in AECOPD, the observed attenuation of systemic inflammation represents a potentially important therapeutic benefit of the combined approach. These objective improvements in lung function, blood gas parameters, and inflammatory markers collectively provide stronger evidence for the efficacy of the combined therapy than subjective symptom-based measures alone.

This study shows that, compared with Ma Ren Pill alone, the combined therapy may be associated with lower posttreatment constipation symptom scores, respiratory symptom scores, constipation resolution time, hospitalization duration, PCO₂, TNF-*α*, IL-8, and CRP, alongside higher PEF, FEV₁, FEV₁/FVC, PO₂, and GQOLI-74 scores. These findings add observational evidence that indirect moxibustion plus Ma Ren Pill may be linked to concurrent improvements in gastrointestinal and respiratory outcomes in this patient population (22–24). The consistency of improvements across multiple objective domains-pulmonary function, blood gas exchange, and inflammatory biomarkers-strengthens the plausibility of a genuine treatment effect, although causal inference remains limited by the retrospective design. Additionally, the overall efficacy of the combined indirect moxibustion therapy is higher (*p* < 0.05). It should be noted that “total effective rate” is a subjective composite measure based on physician assessment and thus should be interpreted cautiously; the primary conclusions are derived from objective indicators such as lung function and inflammatory markers. The main reasons for the above results stem from Ma Ren Pill, which is composed of Cannabis Fructus, Persicae Semen, Rhei Radix et Rhizoma, and other herbs. Modern pharmacological evidence supports its regulation of aquaporin expression, intestinal smooth muscle contractility, and anti-inflammatory pathways [12, 33]. Furthermore, this therapy may inhibit airway inflammation by regulating vagal nerve tension, improving intestinal peristalsis, and through the meridian connection between the lungs and the large intestine (8–11, 25–30). Notably, the magnitude of improvement in objective inflammatory markers (TNF-*α*, IL-8, and CRP) in the observation group suggests that the combination therapy may exert a systemic anti-inflammatory effect beyond that achievable with Ma Ren Pill alone. This is particularly relevant given that persistent systemic inflammation is a key driver of COPD progression and acute exacerbation. The concurrent improvement in both pulmonary function and inflammatory profiles is mainly due to the fact that enhanced intestinal motility reducing endotoxin translocation and subsequent systemic inflammation, thereby alleviating airway inflammation and improving respiratory mechanics. The observed concurrent improvements in constipation and respiratory parameters are consistent with hypothesized gut–lung axis interactions, though direct biological evidence (e.g., microbiome or metabolomic data) was not collected in this study.

Our findings suggest that the combination of indirect moxibustion and Ma Ren Pill is closely related to the effects of bowel regulation, Yang-warming, and anti-inflammatory modulation. It may improve AECOPD and constipation. The main reason for this phenomenon can be attributed to the fact that Ma Ren Pill reduces intestinal toxin absorption and inhibits the NF–κB pathway, thereby decreasing pro-inflammatory cytokines (e.g., TNF-*α* and IL-8); concurrently, indirect moxibustion enhances diaphragmatic dynamics and optimizes ventilation/perfusion ratios through autonomic regulation and the anti-inflammatory properties of Artemisia argyi essential oils. From a mechanistic perspective, the objective improvements in PO₂ and PCO₂ suggest that indirect moxibustion may facilitate diaphragmatic excursion and improve alveolar ventilation, likely through vagal modulation and enhanced respiratory muscle function. The reduction in inflammatory cytokines further suggests that the combined therapy may interrupt the vicious cycle of systemic inflammation and respiratory compromise, hallmarks of severe AECOPD. Regarding safety, it is important to emphasize that all adverse event data were retrospectively identified from electronic medical records rather than prospectively monitored with standardized questionnaires or active surveillance. With this limitation in mind, the documented adverse events were mild and self-limiting in both groups, with no serious events (grade ≥ 3) reported. The observation group experienced a slightly higher but not statistically significant incidence of local skin erythema and moxa smoke intolerance, which are expected and manageable reactions related to moxibustion therapy. However, because mild or transient events-such as minor skin irritation, occasional nausea, or patient discomfort-may not have been consistently documented in routine clinical records, the absence of reported events should not be interpreted as evidence of no occurrence. Therefore, while the available data suggest an acceptable safety profile for the combined regimen, this conclusion must be interpreted cautiously, and the true incidence of mild adverse events may be underestimated in this retrospective analysis. Crucially, as a retrospective study lacking randomization, treatment selection was susceptible to confounding by clinician preference, patient compliance, or undocumented baseline disparities; thus, the observed improvements should be interpreted strictly as observational associations rather than definitive treatment effects, and residual confounding cannot be ruled out.

Several limitations warrant caution. First, because this was a retrospective study, group allocation was based on historical treatment records rather than randomization. Although baseline characteristics were comparable, residual confounding from unmeasured factors (e.g., clinician preference, patient tolerance for moxibustion) cannot be excluded, and the findings should be interpreted as associative rather than causal (23, 24). Second, the single-center, small sample size limits generalizability. Third, constipation assessment relied on subjective symptom scales without objective measures such as colon transit time, and the 2-month follow-up precluded evaluation of long-term efficacy or safety. Fourth, adverse event data were extracted from medical records without prospective standardized monitoring, potentially leading to underreporting of mild discomfort. Future validation via prospective, randomized, controlled trials with longer follow-up, objective outcome measures, and mechanistic biomarkers (e.g., gut microbiota, inflammatory signaling pathways) is required before this combined regime can be considered for broader clinical application.

## 5 Conclusion

In summary, for patients with AECOPD and constipation, the combined intervention of indirect moxibustion and Ma Ren Pill was associated with improvements in constipation, respiratory symptoms, pulmonary function, blood gas parameters, and systemic inflammatory status, and may effectively improve patients’ quality of life with an acceptable safety profile.

## Data Availability

The original contributions presented in the study are included in the article/supplementary material, further inquiries can be directed to the corresponding author.
